# First report of the complete mitochondrial genome and phylogenetic analysis of *Aphrodita australis* (Aphroditidae, Annelida)

**DOI:** 10.1080/23802359.2019.1692712

**Published:** 2019-11-20

**Authors:** Zongxing Wang, Xiuqi Li, Zongjun Xu, Hongzhan Liu, Yanan Wang, Fengrong Zheng

**Affiliations:** a Marine Ecology Research Center of First Institute Oceanography, Ministry of Natural Resources, Qingdao, PR China;; b Shandong Freshwater Fisheries Research Institute, Jinan, PR China;; c Marine College of Shandong University, Weihai, PR China

**Keywords:** *Aphrodita australis*, complete mitochondrial genome, Aphroditidae

## Abstract

In this study, the complete mitochondrial genome of the *Aphrodita australis* was sequenced. The complete mitochondrial genome was circular and 15,288 bp in length, consisted of a typical set of 13 protein-coding genes (PCGs), 2 ribosomal RNA (rRNA) genes, 22 transfer RNA (tRNA) genes, and 1 non-coding control region. All these genes are in the heavy strand. The non-coding control region is 672 bp in length, and located between tRNA-Ser and tRNA-Leu. The overall nucleotides base composition of the heavy strand is 31.02% for A, 22.76% for C, 12.49% for G, and 33.73% for T, with a slight A + T-rich feature (64.75%). All of the PCGs begin with ATG as their start codon and the *cox 3*, *cytb* and *nad 3* are terminated with TAA, *atp8*, *nad4*, *nad 4l*, and *nad 6* are terminated with TAG, while others are terminated by incomplete codon T. Seen from the phylogenetic tree, *A. australis* has a more close relationship with *Goniada japonica* than other species.

*Aphrodita australis* belongs to the family Aphroditidae, with dorsoventrally flattened bodies (Fauchald 1977), brilliantly shining and splendidly iridescent-hair, commonly known by the name of the sea-mouse (William 1865). *Aphrodita australis* live at depths of 70 m and shallower (Andrew 1998), very common in the Northeast Pacific, Japan, and China coast (Izuka 1912; Lei and Sun 2008; Xu et al. 2012; Christopher et al. 2016; Panchenko and Pushchina 2019). Genetic studies on *A. australis* are limited and sparse, hence analysis based on the complete mitochondrial genome provides more useful information on classification and help to better understand the phylogenetic status of this species in Polychaeta. In order to contribute to genetic research, we sequenced its complete mitochondrial genome using the next-generation sequencing (NGS) techniques strategy (Wang et al. 2015).

In this study, *A. australis* was sampled from Jiaozhou Bay (36.05°N, 120.27°E), Shandong Province of China, during January 2019. Specimen (voucher no. FIO201901001) was deposited in the Biodiversity Lab of the First Institute of Oceanography, Ministry of Natural Resources.

The complete mitochondrial genome of *A. australis* (GenBank accession no. MN334532) is 15,288 bp in length, with a A-T content of 64.75%, containing 13 protein-coding genes (PCGs), 2 ribosomal RNA genes (12SrRNA and 16S rRNA), 22 transfer RNA genes (tRNA), and 1 non-coding control region (D-loop). The 13 PCGs correspond to the genes *cox1*, *cox 2*, *cox 3*, *atp 6*, *atp 8*, *cytb*, *nad 1*, *nad 2*, *nad 3*, *nad 4*, *nad 4l*, *nad 5*, and *nad 6*. All these genes are in the heavy strand. The two rRNA and 22 tRNA genes are distributed in the heavy strand, too. The overall nucleotides base composition of the heavy strand is 31.02% for A, 22.76% for C, 12.49%for G, and 33.73% for T, with a slight A + T-rich feature (64.75%). All PCGs begin with ATG. The *cox 3*, *cytb*, and *nad 3* are terminated with TAA, *atp 8*, *nad 4*, *nad 4l*, and *nad 6* are terminated with TAG, while the others are terminated by incomplete codon T. The longest one is *nad 5* gene (1699 bp) among the PCGs and the shortest is *atp 8* gene (153 bp). The non-coding control region (D-loop) is 672 bp in length and is located between RNA-Ser(TGA) and tRNA-Leu (TAA). The rRNA genes, 12S rRNA (804 bp), and 16S rRNA (1255 bp) are located between the *tRNA-Met(CAT)* and *tRNA-Leu(TAG)* genes, and are further separated by the *tRNA-Val(TAC)* gene.

To explore the phylogenetic relationship of Polychaeta and the status of *A. australis*, a phylogenetic tree was constructed based on the maximum likelihood by RAxML version 8.1.5 (Heidelberg, Germany) (Alexandros 2006) analysis using the 13 PCGs of mitochondrial genomes of *A. australis* and the other 18 species of Polychaeta (Figure 1). This is the first report of the complete mitochondrial genome of family Aphroditidae. But in Order Phyllodocida, there have been other 13 recorded complete mitochondrial genome. According to the phylogenetic analysis, *A. australis* has a more close relationship with *Goniada japonica* than other species.

**Figure 1.**
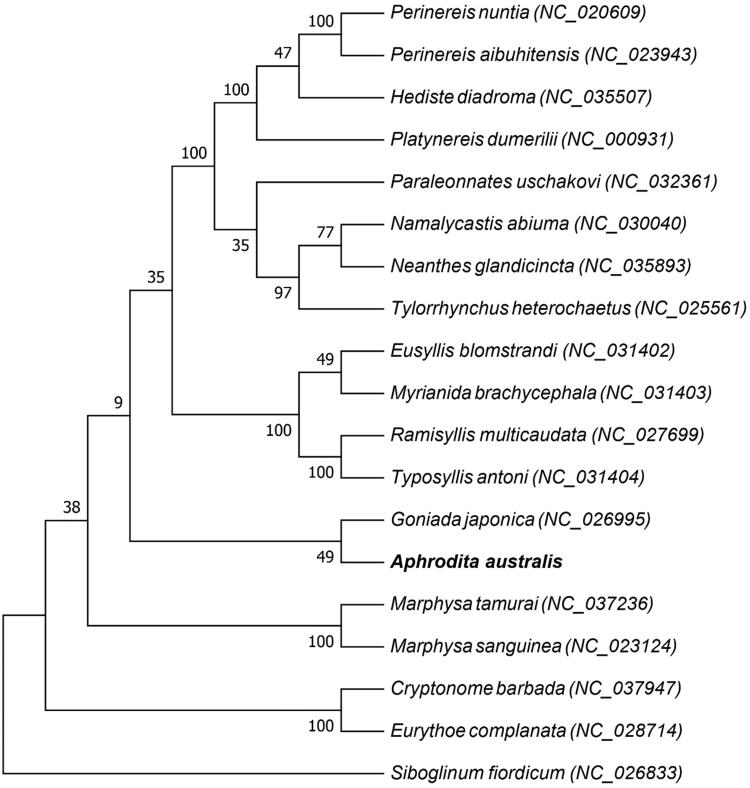
Maximum likelihood tree based on protein coding genes of mitochondrial genomes of *Aphrodita australis* (MN334532) and *Cryptonome barbada* (NC_037947), *Marphysa tamurai* (NC_037236), *Neanthes glandicincta* (NC_035893), *Hediste diadroma* (NC_035507), *Paraleonnates uschakovi* (NC_032361), *Myrianida brachycephala* (NC_031403), *Typosyllis antoni* (NC_031404), *Eusyllis blomstrandi* (NC_031402), *Namalycastis abiuma* (NC_030040), *Eurythoe complanata* (NC_028714), *Ramisyllis multicaudata* (NC_027699), *Goniada japonica* (NC_026995), *Tylorrhynchus heterochetus* (NC_025561), *Perinereis aibuhitensis* (NC_023943), *Marphysa sanguinea* (NC_023124), *Perinereis nuntia* (NC_020609), *Platynereis dumerilii* (NC_000931), and *Siboglinum fiordicum* (NC_026833) is used as outgroup.
